# The Value of Rectal Lavage and Irrigation

**Published:** 1898-12

**Authors:** 


### THE VALUE OF RECTAL LAVAGE AND IRRIGATION.

Dr. Ramon Guiteras, in the *Post-Graduate* for August, 1898, has a very interesting paper on rectal lavage and irrigation, in which he says that rectal lavage and irrigation are practically the same. They both consist in washing out the lower bowel. Lavage usually implies simply washing out the rectum by injecting fluids, whereas irrigation generally carries the idea of watering the inside of the gut, or, in other words, a better graduated method of bringing the fluid in contact with the inside of the rectum continuously during each treatment. This method has been known from time immemorial, and there is nothing new or original in it, excepting perhaps in the technique which will here be described, and the description of the relations of the tissues and organs of the pelvis to one another.

Rectal irrigation consists in allowing pure water or a medicated fluid to run into the bowel and out again for the purpose of cleansing it, or of treating pathological conditions either in the gut itself or in the neighboring tissues or organs.

As to the various fluids used in rectal irrigation, the author has divided them into bland and medicinal: *Bland*—Hot water at a temperature of from 100° to 120°; hot saline solution (one drachm to one quart) at the same temperature; hot flaxseed tea (flaxseed one drachm, water one quart; boil and strain). *Medicinal*—Solution of carbolic acid (half drachm to one pint) $=\frac{1}{250}$; solution of boric acid (tablespoonful to one pint) $=\frac{1}{30}$; solution of permanganate of potassium, $\frac{1}{2000}$; solution of nitrate of silver, $\frac{1}{2000}$; solution of bichloride of mercury, $\frac{1}{10000}$.

In conclusion Dr. Guiteras says:

1. think that in rectal hydrotherapy by means of irrigations we have an extensive field for work and observation.

2. That the best results are obtained in cases where there are distinct pathological lesions in the rectum or lower bowel.

3. That the diseases of the genital tract, both in the male and in the female, are far more benefited by this treatment than is generally supposed.

4. That the influence of these irrigations on the secretion of the kidney, as shown in this article, is of the greatest importance, and should be tried in all cases where other means now in vogue have proved unsuccessful.

5. That the effects of this procedure on the circulation seem to be so stimulating and so opposed to the conditions of shock that it would be advisable to employ it in cases of severe operation where there have been or there is expected to be a considerable loss of blood, as a preventive measure.—*Medical Age*.

